# The Analytical and Clinical Validity of the pfSTEP Digital Biomarker of the Susceptibility/Risk of Declining Physical Function in Community-Dwelling Older Adults

**DOI:** 10.3390/s23115122

**Published:** 2023-05-27

**Authors:** Alexander Schoenfelder, Brad Metcalf, Joss Langford, Afroditi Stathi, Max J. Western, Melvyn Hillsdon

**Affiliations:** 1 Institute for Data Science and AI, University of Exeter, Exeter EX4 4QJ, UK; a.schoenfelder@exeter.ac.uk; 2 Sports and Health Sciences, University of Exeter, Exeter EX1 2LU, UK; b.metcalf@exeter.ac.uk (B.M.); j.langford@exeter.ac.uk (J.L.); 3 Activinsights Ltd., Huntingdon PE28 0NJ, UK; 4 School of Sport, Exercise and Rehabilitation Sciences, University of Birmingham, Birmingham B15 2TT, UK; a.stathi@bham.ac.uk; 5 Department of Health, University of Bath, Bath BA2 7AY, UK; mjw68@bath.ac.uk

**Keywords:** physical activity, accelerometer, biomarker, step count, stepping volume, stepping rate, walking, analytical validity, clinical validity, verification

## Abstract

Measures of stepping volume and rate are common outputs from wearable devices, such as accelerometers. It has been proposed that biomedical technologies, including accelerometers and their algorithms, should undergo rigorous verification as well as analytical and clinical validation to demonstrate that they are fit for purpose. The aim of this study was to use the V3 framework to assess the analytical and clinical validity of a wrist-worn measurement system of stepping volume and rate, formed by the GENEActiv accelerometer and GENEAcount step counting algorithm. The analytical validity was assessed by measuring the level of agreement between the wrist-worn system and a thigh-worn system (activPAL), the reference measure. The clinical validity was assessed by establishing the prospective association between the changes in stepping volume and rate with changes in physical function (SPPB score). The agreement of the thigh-worn reference system and the wrist-worn system was excellent for total daily steps (CCC = 0.88, 95% CI 0.83–0.91) and moderate for walking steps and faster-paced walking steps (CCC = 0.61, 95% CI 0.53–0.68 and 0.55, 95% CI 0.46–0.64, respectively). A higher number of total steps and faster paced-walking steps was consistently associated with better physical function. After 24 months, an increase of 1000 daily faster-paced walking steps was associated with a clinically meaningful increase in physical function (0.53 SPPB score, 95% CI 0.32–0.74). We have validated a digital susceptibility/risk biomarker—pfSTEP—that identifies an associated risk of low physical function in community-dwelling older adults using a wrist-worn accelerometer and its accompanying open-source step counting algorithm.

## 1. Introduction

The use of wearable sensors, including accelerometers, to estimate the number of daily steps and their cadence, has become ubiquitous in research [1]. Their ability to objectively and unobtrusively obtain multi-day, 24/7 recordings of stepping in free-living conditions can provide insights not obtainable within the constraints of the laboratory, such as detailed distributions of step counts, stepping durations, and cadences [2,3,4,5].

A higher stepping volume (the number of steps counted during an interval of time, e.g., steps/day) is associated with reduced all-cause mortality and cause-specific mortality [1,6,7,8,9], as well as a lower risk of chronic disease [10] including cardiovascular disease [11].

Meeting a daily stepping goal through slower-paced walking is qualitatively different from achieving the same daily steps through faster-paced walking. Investigations of the association between an accelerometer-derived stepping volume and rate (the cadence at which steps were accumulated, e.g., 62 steps/min) with health outcomes provide equivocal findings. A higher stepping volume and rate have been shown to be jointly associated with lower hospitalisation and all-cause mortality in older adults [12]. Stepping at a faster pace has also been associated with all-cause, cancer, and cardiovascular morbidity and mortality even when adjusting for the daily stepping volume [13], and it has been suggested that the stepping rate may be of greater importance for cardiometabolic risk reduction than total stepping volume [14]. Furthermore, the association of total stepping volume with all-cause dementia [15] and incident diabetes [16] was found to be stronger when steps were accrued at a faster pace. Similarly, a larger proportion of steps at higher stepping rates was associated with a greater risk reduction for diabetes [17]. In contrast, a higher daily stepping volume was associated with lower mortality but stepping rate was not when adjusted for the stepping volume [7,8,18].

The different measurement properties of the systems used to estimate step counts are one possible reason for the uncertainty about the relative importance of stepping volume and rate, because the outputs of different systems are far from interchangeable [19]. The aspects shaping an accelerometer system’s measurement properties include the design of algorithms turning raw data into stepping estimates [20], the construction and duration of variable-length stepping events [21], and the sensor’s wear location (usually hip, thigh, or wrist) [22,23,24,25,26,27,28]. Discrepancies between systems can also be exacerbated at low stepping rates because it is harder to detect steps from weak acceleration signals [5,25,29,30,31]. Likewise, the acceleration is moderated by the setting in which movement occurs. Stepping metrics estimated from the same device can vary depending on whether the data are recorded in an artificial laboratory setting, such as treadmill walking or simulated activities of daily life, or in an authentic free-living setting [32,33].

Epoch-based methods and event-based methods are two different ways to estimate the stepping volume and the rate at which steps were accumulated. An epoch-based method collects and analyses data in predefined, non-overlapping time intervals (epochs). For example, an epoch might be set to last 60 s, and the number of steps taken during that 60-s interval would be recorded. The number of steps taken are divided by the epoch’s duration to estimate the cadence. This approach underestimates the ‘true’ cadence if the epoch includes stationary time or if the start and end of the stepping event spans over two epochs. This is likely to be an issue because it is uncommon for humans to step consistently for a whole minute [34]. An event-based method, on the other hand, records steps in real-time as they occur [3]. When a step is detected, an event is started, and the number of steps taken recorded until the continuous period of stepping comes to an end. The number of steps in the event is still divided by the duration of the event to estimate the cadence. However, this estimate is a more accurate estimate of the true cadence because the stationary time is not included in the variable-length event’s duration.

When measuring stepping, placing accelerometers on the lower body (e.g., the thigh or hip) is generally preferred because the lower limbs are the body parts in contact with the ground and the primary source of movement during stepping. An accelerometer placed on the upper body, such as the wrist, may still capture the motion of the body during stepping, but the signals can be affected by secondary actions (e.g., holding a phone) and may not always reflect whole body movement [25]. The sensor wear location also impacts wear time adherence, which may lead to differences between studies. Periods of missing data due to non-wear can reduce the accuracy of stepping estimates and lead to erroneous estimates of the association between stepping and health outcomes [35,36]. Reduced wear time for hip-worn devices has been attributed to the discomfort and inconvenience they can cause [37] and evidence suggests that wrist-worn systems have higher adherence to wear time protocols in adolescents and adults [1,38,39].

Comfortable, waterproof, single-device sensors worn at the wrist are more likely to maximise wear time, and thus the accuracy of derived estimates, but these measurement systems need to show that the stepping estimates they produce are reliable and fit for purpose. It has been proposed that biomedical technologies, including accelerometers and their algorithms, should undergo rigorous verification as well as analytical and clinical validation—the V3 framework—to confirm their suitability [40]. While digital measures of stepping have been estimated from wrist-worn accelerometers, none have demonstrated that they are fit-for-purpose based on the V3 framework [25,28,41,42,43,44,45,46]. The results from studies that assessed the accuracy of the widely used wrist-worn ActiGraph in free-living settings and with criterion measures obtained in a laboratory found that stepping estimates obtained from the wrist were often in disagreement with those measured at the hip [26,47,48], highlighting the importance of rigorous verification and validation of accelerometer measurement systems. Therefore, the aims of this study were to apply the complete V3 framework by:

1. Selecting a verified wrist-worn measurement system, formed by the GENEActiv accelerometer [49] and its accompanying open-source step counting algorithm.
2. Establishing its analytical validity by measuring the level of concurrent agreement between the GENEActiv wrist system and the activPAL thigh system when worn simultaneously in a sample of older adults.
3. Establishing its clinical validity by measuring the prospective association between repeated measures of daily stepping volume and rate with physical function measured via the Short Physical Performance Battery (SPPB) score [50] in a sample of older adults. The SPPB score is a clinically based measure of physical function associated with all-cause mortality, hospitalisation, future functional decline, and long-term disability [51,52]. Furthermore, the SPPB score is a predictor of frailty phenotypes and geriatric syndromes in community-dwelling older people [53].

We conclude that wrist-measured stepping volume and rate obtained through the verified and analytically and clinically validated GENEActiv measurement system create a viable digital susceptibility/risk biomarker [54] associated with a decreased risk for low physical function in older, community-dwelling adults not suffering from health conditions preventing them from engaging in physical activity.

## 2. Materials and Methods

### 2.1. Verification

This study was conducted with well-established measurement hardware. The acceleration measurement of the GENEActiv has been shown to have excellent intra-device and inter-device reliability [55]. The function of the open-source step counting algorithm was verified both by code inspection and replication in an alternate code base. The analytical reference device for step measurement was the activPAL [56], which has demonstrated an absolute percentage error of 1% when compared to the leading pedometers [57]. The analysis pipeline was regularly tested throughout development, with full records of the package dependencies.

### 2.2. Analytical Validity

#### 2.2.1. Data Source

Data for the evaluation of the analytical validity were obtained from the ‘Digital Assessment of Precise Physical Activity’ (DAPPA) project (funded by the EPRSC, disseminated via the Get A Move On (GAMO) Network (grant ref: EP/N027299/1), Project 532526 Feasibility Funding). The participants were a convenience sample of 56 people over the age of 50, taking part in a study to develop a suite of measures of physical activity. Participants simultaneously wore the GENEActiv wrist-worn and activPAL thigh-worn accelerometers for 7 consecutive days while going about their usual activity. Those with a disability or injury preventing them from engaging in physical activity were excluded. Ethical approval was obtained by the University of Bath’s Research Ethics Approval Committee (SESHES-20/21R1-008). All participants provided written informed consent prior to participation, including consent for their anonymised data to be used for future research.

#### 2.2.2. Processing of Raw Accelerometer Data

The raw accelerometer data from the thigh- and wrist-worn devices were processed to obtain estimates of stepping volume and rate. Both measurement systems used event-based rather than epoch-based approaches to achieve a granular assessment of stepping volume and rate throughout a day, although the event segmentation approaches were different. The thigh-worn devices ran firmware 649 and their raw data were processed with the manufacturer’s proprietary PALbatch desktop software in version 8.11.1.63 [56] using the VANE algorithm. The minimum non-upright period and minimum upright period durations were set to the default of 10 s. The wear time validation algorithm was set to the most stringent option, using the 24-h protocol, which allows a maximum of 4 h of non-wear per day. Wear correction was enabled to automatically correct inverted wear if a participant accidentally attached the device the wrong way round. There is no calibration option in the PALbatch software. The resulting data were exported via the ‘Events (extended)’ and ‘Stepping bouts’ reports to acquire time-stamped strides and variable-length events.

The wrist-worn devices ran firmware version 4.08a. Their raw sensor data were calibrated to remove potential measurement errors, which may result from local gravity or temperature [58], using the GENEAread R package [59]. The calibrated raw data were then processed into stepping metrics using variable-length events with the GENEAread and GENEAclassify packages [60] in versions 2.0.8 and 1.5.1, respectively. The number of valid wear hours on each measurement day was identified separately with the GGIR package version 2.7-1 [61] by analysing the calibrated raw data in 24-h chunks (midnight to midnight). This made it possible to match the 24-h protocol from the thigh-worn system during data quality checking. All the code (in Supplementary Materials) was run with R version 4.1.3 in R Studio 2022.02.3 Build 492 [62].

#### 2.2.3. Data Quality and Aggregation

The processed stepping measurements were quality checked to ensure that only the relevant and reliable observations were included. Only data recorded on valid days, defined as days on which a participant wore both devices for at least 20 h, were analysed. Sedentary or upright events without stepping activity were excluded, as were events with fewer than 10 steps because fewer than 10 consecutive steps may lead to unreliable estimates [63]. The thigh-worn system did not report cadences less than 20 steps/min, possibly because slow stepping produces smaller accelerations, which do not satisfy the minimum acceleration thresholds necessary for a step to be registered [25]. Wrist events with cadences less than 20 steps/min were therefore removed. Similarly, the thigh-worn system does not report cadences greater than 175 steps/min and such events were removed from the wrist data accordingly. Where participants recorded data for more than 7 consecutive days, the additional days were excluded to avoid a potential distortion of the results by cyclical behaviour, such as work-related activity patterns or exercise routines.

For each participant, the event-level stepping estimates were aggregated into daily measures of stepping volume and rate. Total steps (20–175 steps/min) were obtained by summing up a participant’s steps on each valid day.

Total steps were then further categorised into two subsets: ‘non-walking steps’ (20–44 steps/min) and ‘walking steps’ (45–175 steps/min). Stepping below 45 steps/min was not considered walking, because stepping below this threshold tends to consist of less sustained stepping consistent with non-walking behaviours [5].

**Figure 1:**
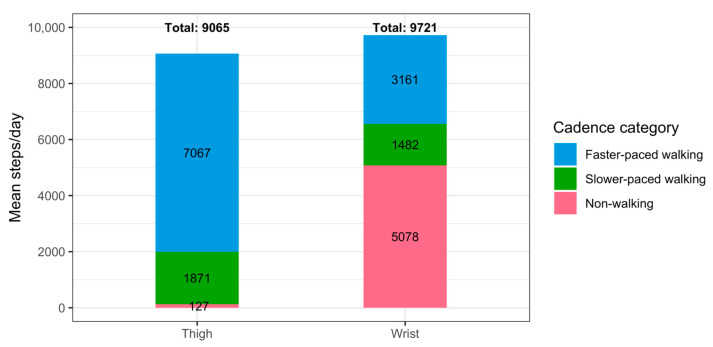
Mean steps per day in the non-walking (20–44 steps/min), slower-paced walking (45–74 steps/min for thigh and 45–76 steps/min for wrist), and faster-paced walking (75–175 steps/min for thigh and 77–175 steps/min for wrist) cadence categories across all participants and days by device.

**Figure A1:**
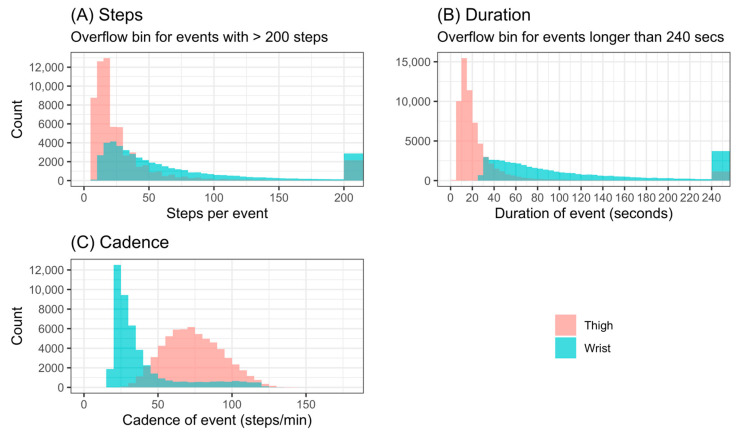
Event-level distributions of (**A**) step count of the event, (**B**) duration of the event, and (**C**) cadence of the event as measured on the thigh and on the wrist.

The ‘walking’ category (45–175 steps/min) was then further divided into two sub-sets representing ‘slower-paced walking’ and ‘faster-paced walking’. A comparison of the distributions of event step counts, durations, and cadences showed that the thigh and wrist systems had different response characteristics due to differences in their processing pipelines (Figure A1). The system-specific cadence thresholds to delineate walking at slower and faster pace were therefore required for a meaningful evaluation of agreement. This was achieved by identifying each system’s median walking cadence (the median cadence of events ≥45 steps/min). For the thigh system, the median walking cadence was 74 steps/min, for the wrist 76 steps/min. Step counts from events below and including the median walking cadence were then summed for each day to obtain slower-paced walking steps and those above the median walking cadence to calculate faster-paced walking steps (Table 1, Figure 1).

### 2.3. Clinical Validity

#### 2.3.1. Data Source

Participants were from the REtirement in ACTion (REACT) study, which was reviewed and approved by the National Health Service (NHS) South East Coast-Surrey Research Ethics Committee (15/LO/2082) and is registered as a completed randomised controlled trial (ISRCTN45627165). All participants provided written informed consent, including the use of anonymised data for future research.

The full study protocol is published in detail elsewhere [64,65]. In short, participants were over the age of 65 and had an SPPB score between four and nine (inclusive). They were also screened for a variety of health-related exclusion criteria before recruitment. Participants were followed for 2 years during which they were asked to wear a wrist-worn accelerometer in a community-dwelling setting for 7 consecutive days at baseline and at 6 months, 12 months, and 24 months after baseline. At each of these four accelerometer recording periods, the participants also completed a laboratory assessment during which their physical function (SPPB score) and other health metrics were recorded.

#### 2.3.2. Processing of Raw Accelerometer Data

Only the wrist-worn measurement system was used in the REACT study and the raw data were processed with the same tools and settings described in Section 2.2.2.

#### 2.3.3. Data Quality and Aggregation of Stepping Metrics

In keeping with the exclusion criteria described in Section 2.2.3, stepping events with fewer than 10 steps, a cadence less than 20 steps/min and greater than 175 steps/min, and measurement days beyond the 7th day were excluded. For each of the four accelerometer recording periods, stepping estimates were only considered valid if the device was worn for at least 18 h/day on at least 6 days/period to maximise the reliability of the habitual physical activity estimates [35]. The 18 h/day wear time limit accounted for the fact that the accelerometers were configured to start recording at 5:00 am on the first measurement day. A minimum of 18 h/day avoided discarding valuable data while producing reliable estimates because it remained considerably more stringent than the recommended wear times [66].

For each valid recording period, the data were aggregated into average daily stepping variables. The aggregation of the stepping measures happened in two stages. First, total steps and the sum of steps accrued during slower-paced and faster-paced walking were calculated for each participant per day. The median walking cadence (62 steps/min in the REACT dataset) was used to delineate slower-paced from faster-paced walking. In the second stage, the daily aggregates were averaged across recording periods, resulting in mean daily step counts for total steps (20–175 steps/min), slower-paced steps (20–62 steps/min), slower-paced walking steps (45–62 steps/min), and faster-paced walking steps (63–175 steps/min) for each of the four recording periods.

### 2.4. Statistical Analysis

#### 2.4.1. Analytical Validity

The wrist-worn system’s analytical validity was determined by assessing the concurrent agreement of its daily stepping estimates with those of the thigh-worn reference standard via the Concordance Correlation Coefficient (CCC) developed by Lin [67] and extended by Carrasco et. al. [68] for longitudinal repeated measurements. This extension expresses the CCC in terms of the variance components of a Linear Mixed Effects Model (LMEM). This accounted for the hierarchical nature of the data by modelling the paired daily stepping estimates as longitudinal replicates separately for each participant (random effects), including the interactions between systems, participants, and recording periods. Separate models were fitted with the cccrm R package version 2.0.3 [69] for total steps and the walking, slower-paced walking, and faster-paced walking subsets to obtain a CCC for each cadence category.

#### 2.4.2. Clinical Validity

The outcome of interest was the change of physical function (SPPB score) over the 24-month follow-up period. Therefore, participants had to provide valid accelerometer and SPPB data for at least two of the four recording periods to be included in the statistical analysis. The independent association between stepping variables and physical function was assessed through LMEMs. All data were analysed at the level of the individual participant and a random intercepts term was included in the model to allow the intercepts to vary for each participant. The covariates included group allocation, site of data collection, age at recruitment, sex, indices of multiple deprivation (IMD) quintile, highest education qualification, perceived general health (SF-36 Score), and the presence of comorbidities (see Table 2 for covariate levels). Data from the control and intervention groups could be analysed together because the effect that the intervention had on SPPB was accounted for by including the group allocation and its respective interaction with time (0, 6, 12, and 24 months) in the model. Longitudinal associations between stepping and SPPB were also modelled by including ‘stepping x time’ interaction terms. Three different models were produced to determine which stepping metric, or combination of them, was best for modelling the SPPB score:

- Model 1: ‘Total steps’ only.
- Model 2: ‘Faster-paced walking steps’ only.
- Model 3: ‘Faster-paced walking steps’ and ‘Slower-paced steps’.

**Table 2:** Characteristics of the study population with regards to the model variables.

	Overall *N* = 651	Male *N* = 217	Female *N* = 434
**Age at recruitment ^1^**	77 (7)	77 (7)	77 (7)
**Baseline physical function (SPPB) ^1^**	8.47 (1.52)	8.60 (1.48)	8.40 (1.55)
IMD quintile ^2^			
1/5 (Most deprived)	69 (11%)	24 (11%)	45 (10%)
2/5	130 (20%)	46 (21%)	84 (19%)
3/5	130 (20%)	46 (21%)	84 (19%)
4/5	136 (21%)	42 (19%)	94 (22%)
5/5 (Least deprived)	186 (29%)	59 (27%)	127 (29%)
**Highest education ^2^**			
Some/All secondary	291 (45%)	91 (42%)	200 (46%)
Some college	171 (26%)	58 (27%)	113 (26%)
All college/Degree	189 (29%)	68 (31%)	121 (28%)
**Comorbidity ^2^**			
None	549 (84%)	182 (84%)	367 (85%)
One or more	102 (16%)	35 (16%)	67 (15%)
**SF-36 General Health Score ^2^**			
Very good/Excellent	99 (15%)	28 (13%)	71 (16%)
Good	310 (48%)	109 (50%)	201 (46%)
Fair/Poor	242 (37%)	80 (37%)	162 (37%)
**Allocation ^2^**			
Control	300 (46%)	98 (45%)	202 (47%)
Intervention	351 (54%)	119 (55%)	232 (53%)
**Site ^2^**			
Bristol/Bath	278 (43%)	89 (41%)	189 (44%)
Birmingham	152 (23%)	58 (27%)	94 (22%)
Exeter	221 (34%)	70 (32%)	151 (35%)

^1^ Mean (SD) ^2^ *N* (%).

Statistical significance of coefficient estimates, for the presence of associations, was defined as *p* < 0.05. All models were fitted in Stata version 17.0 [70] using the ‘mixed’ command. Sensitivity analyses were conducted to confirm the robustness of the LMEM results. This included: (i) fitting the models without health and socio-economic covariates, (ii) the examination of the Control and Intervention groups separately, (iii) the replication of the analysis using mixed effects ordinal logistic regressions (cumulative link models) because SPPB scores lie on a non-equidistant 12-point ordinal scale derived from the sum of three individual four-point scores on an ordinal scale (gait, balance, and sit-to-stand). However, as the 12-point scores were normally distributed, they could be approximated to, and treated as, a continuous scale for the primary LMEM analyses.

### 2.5. Biomarker Description

A detailed analysis of the different measurement systems in this study and those referenced in the prior art made it possible to conceive a simple biomarker that reveals the association of in-community measured steps and physical function (pfSTEP). This biomarker is structured as two integer numbers that represent the average number of slower-paced steps per day, followed by the average number of faster-paced walking steps per day, e.g., (6931; 428). The sum of the two integers is the average total number of steps per day and the separation threshold is the median walking cadence of the population under study.

## 3. Results

### 3.1. Analytical Validity

Of the *N* = 56 participants who provided valid data, 30 (54%) were female and 26 (46%) were male. Their age ranged between 50 and 87 years, with an average age of 64 (±8) years. On average, participants took 9065 (±5104) daily total steps for the thigh-worn reference system and 9721 (±4776) for the wrist-worn system.

The agreement of the thigh- and wrist-worn systems for total daily steps was excellent [71] with a CCC of 0.88 (95% CI 0.83–0.91). The CCC for walking steps and faster-paced walking steps showed a moderate agreement with CCCs of 0.61 (95% CI 0.53–0.68) and 0.55 (95% CI 0.46–0.64), respectively. The CCC for slower-paced walking steps was 0.14 (95% CI 0.02–0.27), indicating a poor agreement for this category with a 95% CI close to zero.

**Figure 2:**
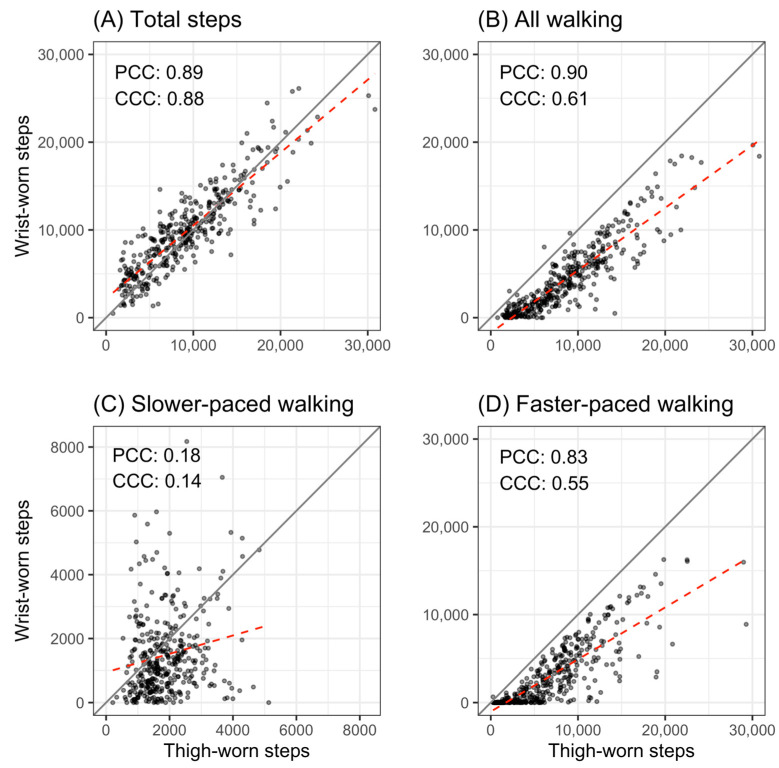
Agreement (Concordance Correlation Coefficient: CCC) and association (Pearson’s Correlation Coefficient: PCC) between the step counts of the thigh- and wrist-worn systems with line of identity (grey) and linear regression line of best fit (red) for total steps (**A**), all walking steps (**B**), slower-paced walking steps (**C**), and faster-paced walking steps (**D**). Each point represents 1 day.

Figure 2 further illustrates the precision and accuracy dimensions of the CCC. The pairs of daily total steps estimated by the two systems were similar (Plot A). A given thigh step count tended to correspond to a similar wrist step count and vice versa, meaning that the measured linear relationship was close to what would be observed in the presence of perfect agreement. The thigh-worn system consistently recorded higher step counts for walking (Plot B). Considerably different thigh and wrist step counts corresponded to each other for slower-paced walking and the true linear relationship between thigh- and wrist-worn measurements at slower-paced walking was far from the theoretical relationship for perfect agreement (Plot C). For faster-paced walking, the agreement was weaker for higher step counts and on many days the thigh-worn reference system recorded considerably more steps than the wrist-worn system (Plot D). The thigh-worn system also consistently reported more faster-paced walking steps than the wrist-worn system.

**Figure A2:**
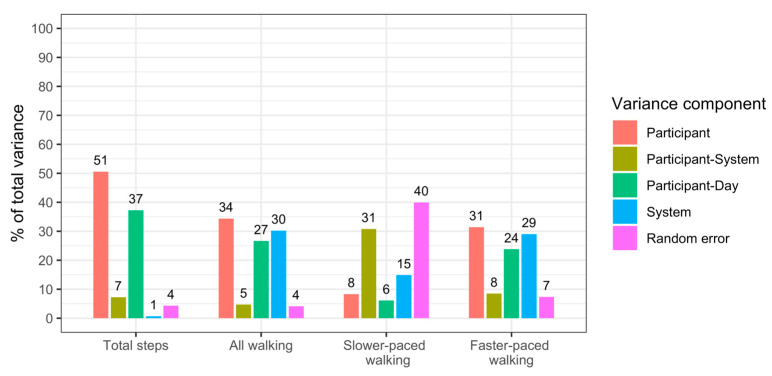
Proportions of variance explained by the variance components from each of the four models fitted separately for total steps (20–175 steps/min), all walking steps (45–175 steps/min), slower-paced walking steps (45–74 steps/min for thigh and 45–76 steps/min for wrist), and faster-paced walking steps (75–175 steps/min for thigh and 77–175 steps/min for wrist).

The variance components from the LMEMs (Figure A2) showed that most of the observed differences between the thigh- and wrist-measured total steps were attributable to the participants (51% of total variance) and the variation of their behaviour on different days (37%), while the measurement systems were the source of little variation (1%). However, when step counts were categorised into slower-paced and faster-paced walking, the measurement systems were a much larger source of variation, contributing between 15% and 30% of the total variance.

### 3.2. Clinical Validity

Of the *N* = 777 participants who took part in the REACT trial, 651 (83.78%) provided both valid accelerometer data and completed the physical function tests on at least two of the four recording periods (10.42% of participants provided data from two periods, 28.44% from three, and 44.92% from all four). Consequently, the model coefficients and goodness-of-fit metrics were derived from stepping activity collected from 15,374 measurement days across 2227 participant-recording periods. On average, each participant provided 6.9 days of accelerometer data from 3.6 recording periods. The age at recruitment ranged from 65 to 98 years. Table 2 presents the characteristics of the study population, resulting from randomisation via a minimisation algorithm, which balanced groups by study site, age group, gender, and initial functional ability [64,72].

Daily total steps and step counts in the cadence-specific categories declined at each follow-up. The proportion of non-walking steps increased over time while the proportion of slower- and faster-paced walking declined (Table 3).

More total steps were consistently associated with better physical function (higher SPPB scores) at all three follow-ups compared to baseline and the association became stronger over the 2-year period. After 24 months, an increase of 1000 daily total steps was associated with a physical function increase of 0.21 points on the 12-point SPPB scale compared to the baseline (Table 4, Model 1).

A higher number of faster-paced walking steps was also consistently associated with better physical function and the association became stronger over time. An additional 1000 daily faster-paced walking steps were associated with a physical function increase of 0.69 points at the 24-month follow-up compared to the baseline (Table 4, Model 2).

Additionally controlling for total steps attenuated the association but it remained present at all time points. An increase by 1000 slower-paced steps (20–62 steps/min) was associated with a physical function increase of 0.13 points while 1000 additional faster-paced walking steps (63–175 steps/min) were associated with an increase of 0.53 points compared to the baseline (Table 4, Model 3).

In relation to the study population’s mean baseline activity levels (Table 3)—which are typical for older adults [9]—1000 additional steps represented a daily physical activity increase of 17% total steps, 20% slower-paced steps, 172% slower-paced walking steps, or 112% faster-paced walking steps.

Sensitivity analyses showed that the health and socio-economic covariates did not alter the presence of the reported associations (Table A1). The results remained the same when the models were replicated as ordinal logistic regressions with mixed effects (Table A2). Separate models of Control and Intervention group data produced comparable results that were also in line with the primary analysis (Table A3 and Table A4).

## 4. Discussion

The aims of this study were to verify and evaluate the analytical and clinical validity of a wrist-worn system for estimating the stepping volume and rate in community-dwelling adults. The results showed that verified, processed data on stepping volume and rate had a high level of agreement with total steps and an acceptable level of agreement with faster-paced walking steps, when directly compared with a thigh-based reference standard in a sample of community-dwelling adults aged over 50.

Direct comparisons with other studies reporting the analytical validity of step counting algorithms, processing data from wrist-worn devices, is challenging due to differences in methodology. The primary challenge is the absence of a true gold standard to classify stepping volume and rate in free-living settings where a direct observation is not feasible. For this reason, most validity studies [44,46,73,74,75,76] are limited to laboratory settings or semi-supervised conditions involving simulated outdoor stepping situations where direct observation is possible for short periods. The performance of step counting algorithms validated under such conditions is poor when they are applied to free-living situations [77,78]. The absence of a gold standard measure prevents the assessment of criterion validity, and it has been proposed that the term ‘reference standard’ be used in situations when the best available method is being used rather than a gold standard [79]. To our knowledge, only one study has assessed the analytical validity of a wrist-worn system in community-dwelling adults using the thigh-worn activPAL as the reference measure [28]. In a sample of *N* = 713 (aged 45 ± 10 years), participants wore both accelerometers together for 7 days. A greater number of daily total steps were recorded for the wrist-worn system compared to the thigh-worn system, a finding similar to the current study. In addition, consistent with the current study, the wrist-worn system had a high level of agreement with daily total steps and a lower level of agreement with faster-paced walking steps (which Maylor et al. [28] defined as >100 steps/min). The level of agreement for slower-paced stepping was not described. As reported in the current study, Maylor and colleagues also comment that the between-accelerometer differences in faster-paced walking steps may be largely due to the reference measure not reliably capturing slower-paced non-walking steps, rather than an error in the wrist-worn system. This again highlights the problem of the absence of a true gold standard criterion measure when assessing a wide range of cadences in free-living settings. In older populations, where the proportion of daily slower-paced steps to total steps is likely to be higher, underestimating slower-paced stepping could be particularly problematic [80].

In the clinical validity study in community-dwelling older adults with a mean age of 77 (±7) years, both faster-paced walking steps (63–175 steps/min) and all other slower-paced steps (20–62 steps/min) were independently associated with higher physical function. The model with both the number of faster-paced walking steps and slower-paced steps was a better fit than models with just total steps (20–175 steps/min) or just faster-paced walking steps. Even if total steps are quite low, if they are mostly faster-paced walking steps, the risk of reduced function is lower than it would be for a higher total number of entirely slower-paced steps.

**Figure A4:**
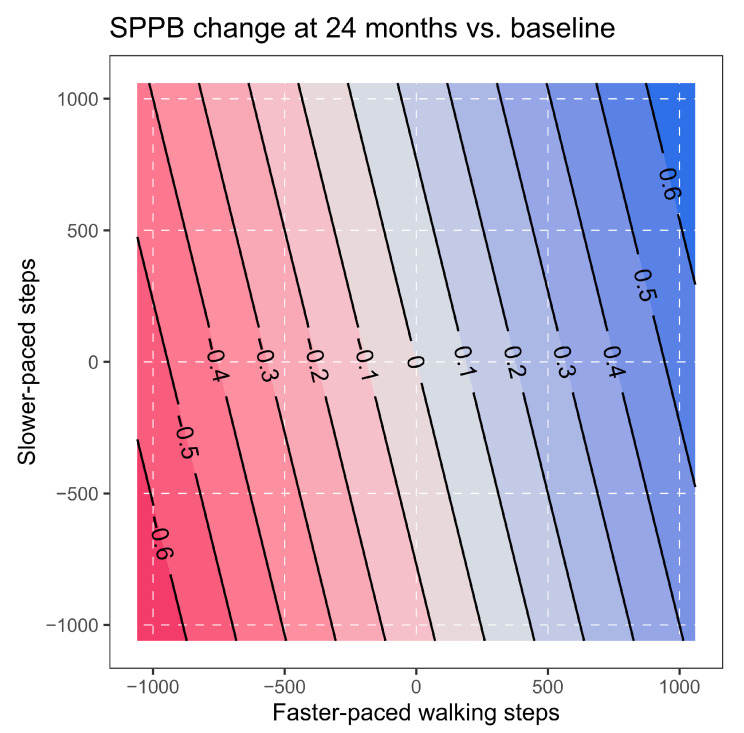
Estimated changes in the SPPB score, at 24 months compared to baseline, with changes in slower-paced steps and faster-paced walking steps.

Over a 2-year period, total steps, slower-paced steps, and faster-paced walking steps fell by 944, 708, and 236 steps/day, respectively, in the combined control and intervention group dataset from the REACT trial. These declines in stepping could potentially lead to a decrease of 0.22 in SPPB score ([0.708 × 0.13] + [0.236 × 0.53]). Consequently, if older people merely retained their baseline stepping level, they could potentially prevent a 0.22 decrease in the SPPB score. However, a meta-analysis of intervention studies has shown that an increase of approximately 1000 steps/day is possible [81]. If achieved in this population (e.g., 500 extra faster-paced walking steps/day and 500 extra slower-paced steps/day), the SPPB score would be expected to increase by 0.33 ([0.500 × 0.53] + [0.500 × 0.13]). Alternatively, if an extra 500 faster-paced walking steps/day were achieved and 500 slower-paced steps/day were replaced with faster-paced walking steps, the SPPB score could increase by 0.47 ([1.000 × 0.53] − [0.500 × 0.13]), a reversal of the expected age-related decline in physical function as well as being a clinically meaningful change [82,83,84]. Increases of 1000 steps/day would not only increase the physical function but also reduce the risk of all-cause mortality and cardiovascular disease morbidity and mortality [1]. Additional examples of changes in slower-paced steps and faster-paced walking steps and the estimated changes in the SPPB score can be found in Figure A4.

To the best of our knowledge, there are no studies that have assessed the clinical validity of accelerometer-derived stepping metrics and objective measures of physical function. More specifically, there are no studies of the association between the changes in stepping volume and rate with changes in physical function. Despite this, our results for daily total steps are consistent with the many prospective cohort studies that consistently show that higher volumes of daily stepping are associated with reduced risk of mortality and chronic disease [1,11,13].

However, there remains uncertainty about whether stepping at a faster pace is associated with health benefits, independent of the total steps taken per day. In this study, increases in faster-paced walking steps were more strongly associated with physical function than increases in slower-paced steps. Different devices, their wear location, and step detection methods can lead to different estimates of stepping rate. The lowest stepping rate able to be reliably detected also varies between devices [25]. Inevitably, this will lead to the misclassification of stepping rates, especially in older adult populations where slow stepping rates are most prevalent. Nevertheless, different devices attached to the same body part and using the same processing algorithm can reduce the differences between device outputs, at least for ‘average-paced’ walking [46]. Some studies calculate the stepping rate using an epoch method while others, including this study, use an event-based method (identify a variable-length stepping event, count the number of steps in the event, and divide by the duration of the event). It has been reported that the epoch method underestimates the ‘true’ stepping rate because it includes periods of standing as well as stepping into one and the same epoch [85]. In addition, event-based methods may be better placed to establish the independence of stepping rate because the stepping rates estimated from epoch methods are more correlated with total steps [86]. In addition, some studies only computed the stepping rates for epochs ≥2 min and cadences ≥60 steps/min [8], whereas others computed cadences as low as 1–39 steps/min [13], even though the device used was not validated for such low cadences.

Declines in physical function are insidious and start at a point when traditional measures of physical function, such as the SPPB score, would likely return ‘normal’ values despite the function already being in decline. We show that the wrist-worn system evaluated in this paper is fit for purpose to obtain a digital biomarker for the early detection of people’s susceptibility or risk of decline in physical function and can be measured remotely at a time when people still have a reserve of function sufficient to alter their trajectory towards low function and frailty. A meta-analysis of interventions [81] has shown that the level of change required to preserve or improve the function identified in this study can be achieved and would also be accompanied by a significant reduction in the risk of chronic disease and all-cause mortality [1].

The GENEActiv wrist-worn system used in this study achieves a high wear time compliance in a variety of populations, is low burden for the wearer, and is proven to be easily deployable in a wide range of applications. The pfSTEP biomarker can be derived from the GENEActiv raw (sensor-level) acceleration data using standard approaches and the open-source GENEAcount algorithm. The continuous measurement of body movement from the wrist is fully aligned with the intended utility of the pfSTEP biomarker (assessing physical function through stepping volume and rate). The representation of the biomarker as two integers retains the intuitive simplicity and usability of steps for both individuals and clinicians, while providing a much richer outcome to support decision-making.

A strength of this study is the methodical approach to the V3 process [40] for assessing how fit for purpose the measures of stepping volume and rate obtained from the wrist are as a digital biomarker of susceptibility/risk for low physical function in older, community-dwelling adults. In addition, the analytical validity was assessed in a real-world setting over several days, better reflecting daily living values of stepping rates compared to laboratory estimates of gait speed [87]. The repeated measures of both exposure and outcome measures are a real strength of this clinical validity study, along with the large sample of community-dwelling older adults.

A major strength of the current study is that it has collected measures of both the exposure (stepping) and the health outcome (physical function) at four time points over a 2-year period. The wholly longitudinal nature of these data allows for the analysis of dynamic associations, rather than the static associations afforded by cross-sectional designs. Dynamic associations in this analysis are represented by the ‘stepping x time’ interaction term, which describe to what degree time-related changes in the SPPB score are associated with time-related changes in stepping. A more common approach in longitudinal studies is to measure stepping once—at the baseline timepoint—and measure physical function at baseline and follow up. The absence of repeated measures of the exposure in such studies would be a major limitation in ageing populations, as this study showed that large decreases in daily total steps, especially at faster-paced walking, occurred over a 2-year period (a reduction of 16% and 26%, respectively). Repeated, longitudinal data are also likely to improve the reliability of associations compared to cross-sectional data as they are less affected by the occurrence of non-typical measures (e.g., a non-representative week of walking/stepping or sub-optimal performance in the SPPB tests). Representing total steps with two different stepping variables of non-overlapping cadence (faster-paced walking steps and slower-paced steps) in the same model makes for an intuitive interpretation of the model coefficients and reduces the level of collinearity between predictors. If total steps and faster-paced walking steps (a sub-set of total steps) had been entered into the same model, this would have caused a high level of collinearity, which in turn would have increased the uncertainty and decreased the reliability of their respective model coefficients. Furthermore, the ‘total steps’ coefficient would represent the coefficient of ‘slower-paced steps’ with ‘faster-paced walking steps’ already being accounted for in the model.

This study is the first to be methodical in trying to match the processing methods for both systems as much as possible. Future studies of analytical validity in real-world settings would benefit from being more transparent about the differences in the step detection methods to ensure that the measurement systems are not a large source of the variance between the stepping estimates, potentially leading to false conclusions about the accuracy of the system being compared to the reference system.

A major limitation in this study, and any other analytical validity study in free-living settings, was the absence of a true gold standard criterion measure. As a result, differences in the estimates of stepping could not be attributed to a misclassification in one system or the other. However, it has been observed that, in situations where an acceptable reference standard does not exist, clinical validation can provide a significant methodological advance [79]. Furthermore, our analytical and clinical validity studies were restricted to older people, limiting the external validity of the results. Additional studies are required in a broader range of populations to determine how generalisable the results are.

The well-documented challenge of accurately detecting slower-paced stepping [5,25,29,30,31], especially in older people, requires urgent attention to better understand the value of slower-paced stepping in this population. Systematic reviews of the prospective association of stepping measures and health outcomes struggle to harmonise the data for meta-analysis due to the very many differences in the systems used to collect the estimates of stepping measures. With the increasing availability of cloud storage, it is possible to store the raw acceleration data, from which stepping measures are derived, at scale. This would allow future reviews to apply a single processing method to raw acceleration data collected from different devices if the wear location was consistent, the wear time was standardised, and the device outputs were verified. This could improve the precision of estimates of the associations between stepping and health outcomes. 

## 5. Conclusions

We have described and validated a digital susceptibility/risk biomarker—pfSTEP—that identifies the associated risk of a low physical function in community-dwelling older adults using a wrist-worn accelerometer and its accompanying open-source step counting algorithm. Older adults who increase their proportion of faster-paced walking steps reduce their risk of developing low physical function and thereby their risk of premature mortality, frailty, hospitalisation, and falls. The digital pfSTEP biomarker uses real world evidence from a system with proven high usability. It supports continuous measurement outside the confines of the clinic or laboratory environment and enables the remote monitoring of changes in ambulatory activity to identify older adults at risk of developing a low physical function.

## Figures and Tables

**Table 1:** Cadence thresholds for the aggregation of daily stepping and walking.

	Thigh-Worn ^1^	Wrist-Worn ^1^
Total steps	20–175	20–175
Non-walking steps	20–44	20–44
Slower-paced steps	20–74	20–76
Walking steps	45–175	45–175
Slower-paced walking steps	45–74	45–76
Faster-paced walking steps	75–175	77–175

^1^ steps/min.

**Table 3:** Daily means of wrist accelerometer-derived step counts by cadence category. Combined data from Control and Intervention groups. Slower-paced steps are the sum of non-walking steps and slower-paced walking steps.

	Baseline *N* = 608	6 Months *N* = 565	12 Months *N* = 544	24 Months *N* = 504
**Total steps** (20–175 steps/min)	5815 (3186; 100)	5569 (3256; 100)	5250 (3017; 100)	4871 (3039; 100)
**Non-walking steps** (20–44 steps/min)	4342 (2371; 75)	4147 (2386; 74)	3965 (2259; 76)	3776 (2353; 78)
**Slower-paced steps** (20–62 steps/min)	4923 (2640; 85)	4702 (2676, 84)	4470 (2490; 86)	4215 (2570, 87)
**Slower-paced walking steps** (45–62 steps/min)	581 (530; 10)	555 (544; 10)	505 (484; 10)	439 (459; 9)
**Faster-paced walking steps** (63–175 steps/min)	892 (938; 15)	867 (996; 16)	780 (915; 15)	656 (857; 13)
**Mean (SD; % of total).**				

**Table 4:** Comparing models containing ‘total steps’ and ‘faster-paced walking steps’ separately and concurrently, in the prediction of physical function.

	Model 1 Total Steps Only	Model 2 Faster-Paced Walking Steps Only	Model 3 Faster-Paced Walking and Slower-Paced Steps
**Total steps** **(20–175 steps/min)**			
Baseline	0.04 (−0.01–0.08)	N/A	N/A
6 Months	0.07 (0.02–0.12) **	N/A	N/A
12 Months	0.12 (0.07–0.17) ***	N/A	N/A
24 Months	0.21 (0.15–0.26) ***	N/A	N/A
**Faster-paced walking steps** **(63–175 steps/min)**			
Baseline	N/A	0.08 (−0.07–0.23)	0.05 (−0.12–0.13)
6 Months	N/A	0.27 (0.10–0.44) **	0.22 (0.03–0.41) *
12 Months	N/A	0.43 (0.25–0.60) ***	0.35 (0.15–0.55) **
24 Months	N/A	0.69 (0.50–0.88) ***	0.53 (0.32–0.74) ***
**Slower-paced steps** **(20–62 steps/min)**			
Baseline	N/A	N/A	0.03 (−0.03–0.10)
6 Months	N/A	N/A	0.04 (−0.03–0.10)
12 Months	N/A	N/A	0.06 (−0.01–0.13)
24 Months	N/A	N/A	0.13 (0.06–0.20) ***
**Goodness-of-fit**			
AIC	8759	8767	8749
Log-likelihood	−4352	−4356	−4342

Unstandardised coefficient estimates reported per 1000 steps with 95% confidence intervals. All models are adjusted for age, sex, site, allocation to Control or Intervention, SF-36 Score, comorbidities, IMD quintile, and highest education in addition to the stepping variables shown. The reference level for all interactions is baseline. The 6-, 12-, and 24-month coefficients represent how much stronger the coefficient is compared to baseline and need to be added to the baseline coefficient to obtain the total strength of the association at each time point (e.g., 0.04 + 0.21 = 0.25 for total steps at 24 months in Model 1). Only models 2 and 3 are nested. N/A = not applicable (variable not included in model) AIC = Akaike Information Criterion (lower by 2 units is considered a better model). * *p* < 0.05, ** *p* < 0.01, *** *p* < 0.001 (coefficient statistically significantly greater than reference baseline coefficient).

**Figure A3:**
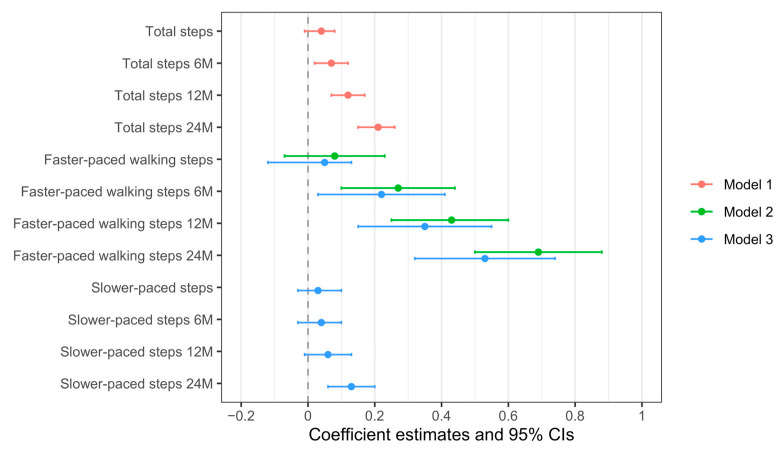
Comparison of coefficient estimates and 95% CIs from the models containing ‘total steps’ and ‘faster-paced walking steps’ separately and concurrently in the prediction of physical function (SPPB score).

## Data Availability

The data presented in this study are available on request from Afroditi Stahti (REACT dataset) and Max Western (DAPPA dataset). The data are not publicly available due to privacy protection.

## Supplementary Materials

The following supporting information can be downloaded at: https://www.mdpi.com/article/10.3390/s23115122/s1, Code S1: R code for the processing, cleaning, analysis, and visualisation of the analytical validity study; Code S2: R code for the processing, cleaning, and visualisation of the clinical validity study; Code S3: Stata code for the analysis of the clinical validity study.

## Appendix B

**Table A1:** Comparing models containing ‘total steps’ and ‘faster-paced walking steps’ separately and concurrently, without health and socio-economic covariates, in the prediction of physical function.

	Model 1b Total Steps Only	Model 2b Faster-Paced Walking Steps Only	Model 3b Faster-Paced Walking and Slower-Paced Steps
**Total steps** **(20–175 steps/min)**			
Baseline	0.05 (0.00–0.09) *	N/A	N/A
6 Months	0.08 (0.03–0.13) **	N/A	N/A
12 Months	0.13 (0.07–0.18) ***	N/A	N/A
24 Months	0.22 (0.16–0.27) ***	N/A	N/A
**Faster-paced walking steps** **(63–175 steps/min)**			
Baseline	N/A	0.10 (−0.05–0.25)	0.06 (−0.10–0.23)
6 Months	N/A	0.28 (0.11–0.45) **	0.24 (0.05–0.43) *
12 Months	N/A	0.44 (0.26–0.62) ***	0.36 (0.16–0.56) ***
24 Months	N/A	0.72 (0.53–0.91) ***	0.54 (0.33–0.75) ***
**Slower-paced steps** **(20–62 steps/min)**			
Baseline	N/A	N/A	0.04 (−0.02–0.10)
6 Months	N/A	N/A	0.04 (−0.04–0.11)
12 Months	N/A	N/A	0.07 (−0.01–0.14)
24 Months	N/A	N/A	0.14 (0.07–0.21) ***
**Goodness-of-fit**			
AIC	8788	8799	8776
Log-likelihood	−4375	−4381	−4365

Unstandardised coefficient estimates reported per 1000 steps with 95%-confidence intervals. All models are adjusted for age, sex, site, and allocation to Control or Intervention in addition to the stepping variables shown. Reference level for all interactions is the baseline visit. Note that only models 2 and 3 are nested. N/A = not applicable (variable not included in model) AIC = Akaike Information Criterion (lower by 2 units is considered a better model) * *p* < 0.05, ** *p* < 0.01, *** *p* < 0.001 (coefficient statistically significantly greater than reference baseline coefficient).

**Table A2:** Comparing models containing ‘total steps’ and ‘faster-paced walking steps’ separately and concurrently, modelled as ordinal logistic regression with mixed effects, in the prediction of physical function.

	Model 1c Total Steps Only	Model 2c Faster-Paced Walking Steps Only	Model 3c Faster-Paced Walking and Slower-Paced Steps
**Total steps** **(20–175 steps/min)**			
Baseline	0.04 (−0.02–0.10)	N/A	N/A
6 Months	0.12 (0.05–0.19) ***	N/A	N/A
12 Months	0.17 (0.10–0.24) ***	N/A	N/A
24 Months	0.29 (0.21–0.36) ***	N/A	N/A
**Faster-paced walking steps** **(63–175 steps/min)**			
Baseline	N/A	0.09 (−0.11–0.29)	0.06 (−0.17–0.28)
6 Months	N/A	0.43 (0.20–0.65) ***	0.36 (0.10–0.61) **
12 Months	N/A	0.65 (0.40–0.89) ***	0.55 (0.27–0.82) ***
24 Months	N/A	1.01 (0.74–1.29) ***	0.79 (0.49–1.09) ***
**Slower-paced steps** **(20–62 steps/min)**			
Baseline	N/A	N/A	0.03 (−0.05–0.12)
6 Months	N/A	N/A	0.06 (−0.03–0.15)
12 Months	N/A	N/A	0.08 (−0.02–0.17)
24 Months	N/A	N/A	0.17 (0.07–0.27) **
**Goodness-of-fit**			
AIC	8573	8570	8557
Log-likelihood	−4249	−4247	−4236

Unstandardised coefficient estimates are log-odds-ratios reported per 1000 steps with 95% confidence intervals. All models are adjusted for age, sex, site, allocation to Control or Intervention, SF-36 Score, comorbidities, IMD quintile, and highest education in addition to the stepping variables shown. The reference level for all interactions is the baseline visit. Note that only models 2 and 3 are nested. N/A = not applicable (variable not included in model) AIC = Akaike Information Criterion (lower by 2 units is considered a better model) ** *p* < 0.01, *** *p* < 0.001 (coefficient statistically significantly greater than reference baseline coefficient).

**Table A3:** Comparing models fitted to Control group data only, containing ‘total steps’ and ‘faster-paced walking steps’ separately and concurrently, in the prediction of physical function.

	Model 1d Total Steps Only	Model 2d Faster-Paced Walking Steps Only	Model 3d Faster-Paced Walking and Slower-Paced Steps
**Total steps** **(20–175 steps/min)**			
Baseline	0.04 (−0.03–0.10)	N/A	N/A
6 Months	0.00 (−0.07–0.16)	N/A	N/A
12 Months	0.08 (0.00–0.16) *	N/A	N/A
24 Months	0.19 (0.11–0.27) ***	N/A	N/A
**Faster-paced walking steps** **(63–175 steps/min)**			
Baseline	N/A	0.08 (−0.13–0.29)	0.05 (−0.19–0.29)
6 Months	N/A	0.09 (−0.17–0.35)	0.13 (−0.16–0.42)
12 Months	N/A	0.36 (0.06–0.67) *	0.32 (−0.01–0.66)
24 Months	N/A	0.57 (0.30–0.84) ***	0.45 (0.16–0.74) **
**Slower-paced steps** **(20–62 steps/min)**			
Baseline	N/A	N/A	0.03 (−0.05–0.12)
6 Months	N/A	N/A	−0.03 (−0.13–0.07)
12 Months	N/A	N/A	0.03 (−0.08–0.14)
24 Months	N/A	N/A	0.13 (0.03–0.23) *
**Goodness-of-fit**			
AIC	4026	4032	4027
Log-likelihood	−1989	−1992	−1986

Unstandardised coefficient estimates reported per 1000 steps with 95% confidence intervals. All models are adjusted for age, sex, site, SF-36 Score, comorbidities, IMD quintile, and highest education in addition to the stepping variables shown. The reference level for all interactions is the baseline visit. Note that only models 2 and 3 are nested. N/A = not applicable (variable not included in model) AIC = Akaike Information Criterion (lower by 2 units is considered a better model) * *p* < 0.05, ** *p* < 0.01, *** *p* < 0.001 (coefficient statistically significantly greater than reference baseline coefficient).

**Table A4:** Comparing models fitted to Intervention group data only, containing ‘total steps’ and ‘faster-paced walking steps’ separately and concurrently, in the prediction of physical function.

	Model 1e Total Steps Only	Model 2e Faster-Paced Walking Steps Only	Model 3e Faster-Paced Walking and Slower-Paced Steps
**Total steps** **(20–175 steps/min)**			
Baseline	0.04 (−0.03–0.10)	N/A	N/A
6 Months	0.13 (0.06–0.20) ***	N/A	N/A
12 Months	0.15 (0.08–0.22) ***	N/A	N/A
24 Months	0.22 (0.15–0.30) ***	N/A	N/A
**Faster-paced walking steps** **(63–175 steps/min)**			
Baseline	N/A	0.09 (−0.11–0.30)	0.07 (−0.16–0.30)
6 Months	N/A	0.37 (0.14–0.59) **	0.25 (0.00–0.51) *
12 Months	N/A	0.45 (0.23–0.69) ***	0.35 (0.09–0.60) **
24 Months	N/A	0.82 (0.55–1.09) ***	0.62 (0.31–0.92) ***
**Slower-paced steps** **(20–62 steps/min)**			
Baseline	N/A	N/A	0.02 (−0.06–0.11)
6 Months	N/A	N/A	0.10 (0.00–0.20) *
12 Months	N/A	N/A	0.09 (−0.01–0.19)
24 Months	N/A	N/A	0.14 (0.03–0.24) *
**Goodness-of-fit**			
AIC	4744	4749	4740
Log-likelihood	−2348	−2351	−2342

Unstandardised coefficient estimates reported per 1000 steps with 95% confidence intervals. All models are adjusted for age, sex, site, SF-36 Score, comorbidities, IMD quintile, and highest education in addition to the stepping variables shown. The reference level for all interactions is the baseline visit. Note that only models 2 and 3 are nested. N/A = not applicable (variable not included in model) AIC = Akaike Information Criterion (lower by 2 units is considered a better model) * *p* < 0.05, ** *p* < 0.01, *** *p* < 0.001 (coefficient statistically significantly greater than reference baseline coefficient).

## References

[1] Hall K.S., Hyde E.T., Bassett D.R., Carlson S.A., Carnethon M.R., Ekelund U., Evenson K.R., Galuska D.A., Kraus W.E., Lee I.-M. (2020). Systematic Review of the Prospective Association of Daily Step Counts with Risk of Mortality, Cardiovascular Disease, and Dysglycemia. Int. J. Behav. Nutr. Phys. Act..

[2] Tudor-Locke C., Camhi S.M., Leonardi C., Johnson W.D., Katzmarzyk P.T., Earnest C.P., Church T.S. (2011). Patterns of Adult Stepping Cadence in the 2005–2006 NHANES. Prev. Med..

[3] Granat M.H. (2012). Event-Based Analysis of Free-Living Behaviour. Physiol. Meas..

[4] Granat M., Clarke C., Holdsworth R., Stansfield B., Dall P. (2015). Quantifying the Cadence of Free-Living Walking Using Event-Based Analysis. Gait Posture.

[5] Stansfield B., Hajarnis M., Sudarshan R. (2015). Characteristics of Very Slow Stepping in Healthy Adults and Validity of the ActivPAL3^TM^ Activity Monitor in Detecting These Steps. Med. Eng. Phys..

[6] Kraus W.E., Janz K.F., Powell K.E., Campbell W.W., Jakicic J.M., Troiano R.P., Sprow K., Torres A., Piercy K.L. (2019). Daily Step Counts for Measuring Physical Activity Exposure and Its Relation to Health. Med. Sci. Sports Exerc..

[7] Lee I.-M., Shiroma E.J., Kamada M., Bassett D.R., Matthews C.E., Buring J.E. (2019). Association of Step Volume and Intensity with All-Cause Mortality in Older Women. JAMA Intern. Med..

[8] Saint-Maurice P.F., Troiano R.P., Bassett D.R., Graubard B.I., Carlson S.A., Shiroma E.J., Fulton J.E., Matthews C.E. (2020). Association of Daily Step Count and Step Intensity with Mortality among US Adults. JAMA.

[9] Paluch A.E., Bajpai S., Bassett D.R., Carnethon M.R., Ekelund U., Evenson K.R., Galuska D.A., Jefferis B.J., Kraus W.E., Lee I.-M. (2022). Daily Steps and All-Cause Mortality: A Meta-Analysis of 15 International Cohorts. Lancet Public Health.

[10] Master H., Annis J., Huang S., Beckman J.A., Ratsimbazafy F., Marginean K., Carroll R., Natarajan K., Harrell F.E., Roden D.M. (2022). Association of Step Counts over Time with the Risk of Chronic Disease in the All of Us Research Program. Nat. Med..

[11] Paluch A.E., Bajpai S., Ballin M., Bassett D.R., Buford T.W., Carnethon M.R., Chernofsky A., Dooley E.E., Ekelund U., Evenson K.R. (2023). Prospective Association of Daily Steps with Cardiovascular Disease: A Harmonized Meta-Analysis. Circulation.

[12] Mañas A., del Pozo Cruz B., Ekelund U., Losa Reyna J., Rodríguez Gómez I., Carnicero Carreño J.A., Rodríguez Mañas L., García García F.J., Ara I. (2022). Association of Accelerometer-Derived Step Volume and Intensity with Hospitalizations and Mortality in Older Adults: A Prospective Cohort Study. J. Sport Health Sci..

[13] del Pozo Cruz B., Ahmadi M.N., Lee I.-M., Stamatakis E. (2022). Prospective Associations of Daily Step Counts and Intensity With Cancer and Cardiovascular Disease Incidence and Mortality and All-Cause Mortality. JAMA Intern. Med..

[14] Sumner J., Uijtdewilligen L., Yee A.C.H., Xian S.N.H., Barreira T.V., Sloan R.A., Van Dam R.M., Müller-Riemenschneider F. (2020). Volume and Intensity of Stepping Activity and Cardiometabolic Risk Factors in a Multi-Ethnic Asian Population. Int. J. Environ. Res. Public. Health.

[15] del Pozo Cruz B., Ahmadi M., Naismith S.L., Stamatakis E. (2022). Association of Daily Step Count and Intensity With Incident Dementia in 78 430 Adults Living in the UK. JAMA Neurol..

[16] Garduno A.C., LaCroix A.Z., LaMonte M.J., Dunstan D.W., Evenson K.R., Wang G., Di C., Schumacher B.T., Bellettiere J. (2022). Associations of Daily Steps and Step Intensity with Incident Diabetes in a Prospective Cohort Study of Older Women: The OPACH Study. Diabetes Care.

[17] Cuthbertson C.C., Moore C.C., Sotres-Alvarez D., Heiss G., Isasi C.R., Mossavar-Rahmani Y., Carlson J.A., Gallo L.C., Llabre M.M., Garcia-Bedoya O.L. (2022). Associations of Steps per Day and Step Intensity with the Risk of Diabetes: The Hispanic Community Health Study/Study of Latinos (HCHS/SOL). Int. J. Behav. Nutr. Phys. Act..

[18] Paluch A.E., Gabriel K.P., Fulton J.E., Lewis C.E., Schreiner P.J., Sternfeld B., Sidney S., Siddique J., Whitaker K.M., Carnethon M.R. (2021). Steps per Day and All-Cause Mortality in Middle-Aged Adults in the Coronary Artery Risk Development in Young Adults Study. JAMA Netw. Open.

[19] Welk G.J., Mcclain J., Ainsworth B.E. (2012). Protocols for Evaluating Equivalency of Accelerometry-Based Activity Monitors. Med. Sci. Sports Exerc..

[20] Ellingson L.D., Hibbing P.R., Welk G.J., Dailey D., Rakel B.A., Crofford L.J., Sluka K.A., Frey-Law L.A. (2019). Choice of Processing Method for Wrist-Worn Accelerometers Influences Interpretation of Free-Living Physical Activity Data in a Clinical Sample. J. Meas. Phys. Behav..

[21] Logan G.R.M., Duncan S., Harris N.K., Hinckson E.A., Schofield G. (2016). Adolescent Physical Activity Levels: Discrepancies with Accelerometer Data Analysis. J. Sports Sci..

[22] Rosenberger M.E., Haskell W.L., Albinali F., Mota S., Nawyn J., Intille S. (2013). Estimating Activity and Sedentary Behavior from an Accelerometer on the Hip or Wrist. Med. Sci. Sports Exerc..

[23] Kamada M., Shiroma E.J., Harris T.B., Lee I.-M. (2016). Comparison of Physical Activity Assessed Using Hip- and Wrist-Worn Accelerometers. Gait Posture.

[24] Cooke A.B., Daskalopoulou S.S., Dasgupta K. (2018). The Impact of Accelerometer Wear Location on the Relationship between Step Counts and Arterial Stiffness in Adults Treated for Hypertension and Diabetes. J. Sci. Med. Sport.

[25] John D., Morton A., Arguello D., Lyden K., Bassett D. (2018). “What Is a Step?” Differences in How a Step Is Detected among Three Popular Activity Monitors That Have Impacted Physical Activity Research. Sensors.

[26] Mandigout S., Lacroix J., Perrochon A., Svoboda Z., Aubourg T., Vuillerme N. (2019). Comparison of Step Count Assessed Using Wrist- and Hip-Worn Actigraph GT3X in Free-Living Conditions in Young and Older Adults. Front. Med..

[27] Park S., Toth L.P., Hibbing P.R., Springer C.M., Kaplan A.S., Feyerabend M.D., Crouter S.E., Bassett D.R. (2019). Dominant vs. Non-Dominant Wrist Placement of Activity Monitors: Impact on Steps per Day. J. Meas. Phys. Behav..

[28] Maylor B.D., Edwardson C.L., Dempsey P.C., Patterson M.R., Plekhanova T., Yates T., Rowlands A.V. (2022). Stepping towards More Intuitive Physical Activity Metrics with Wrist-Worn Accelerometry: Validity of an Open-Source Step-Count Algorithm. Sensors.

[29] Lee K.-Y., Macfarlane D.J., Cerin E. (2013). Comparison of Three Models of Actigraph Accelerometers during Free Living and Controlled Laboratory Conditions. Eur. J. Sport Sci..

[30] Sellers C., Dall P., Grant M., Stansfield B. (2016). Validity and Reliability of the ActivPAL3 for Measuring Posture and Stepping in Adults and Young People. Gait Posture.

[31] Bourke A.K., Ihlen E.A.F., Helbostad J.L. (2019). Validation of the ActivPAL3 in Free-Living and Laboratory Scenarios for the Measurement of Physical Activity, Stepping, and Transitions in Older Adults. J. Meas. Phys. Behav..

[32] Feito Y., Garner H.R., Bassett D.R. (2015). Evaluation of ActiGraph’s Low-Frequency Filter in Laboratory and Free-Living Environments. Med. Sci. Sports Exerc..

[33] Takayanagi N., Sudo M., Yamashiro Y., Lee S., Kobayashi Y., Niki Y., Shimada H. (2019). Relationship between Daily and In-Laboratory Gait Speed among Healthy Community-Dwelling Older Adults. Sci. Rep..

[34] Dall P.M., Mccrorie P.R.W., Granat M.H., Stansfield B.W. (2013). Step Accumulation per Minute Epoch Is Not the Same as Cadence for Free-Living Adults. Med. Sci. Sports Exerc..

[35] Dillon C.B., Fitzgerald A.P., Kearney P.M., Perry I.J., Rennie K.L., Kozarski R., Phillips C.M. (2016). Number of Days Required to Estimate Habitual Activity Using Wrist-Worn GENEActiv Accelerometer: A Cross-Sectional Study. PLoS ONE.

[36] Meyer B.M., Depetrillo P., Franco J., Donahue N., Fox S.R., O’Leary A., Loftness B.C., Gurchiek R.D., Buckley M., Solomon A.J. (2022). How Much Data Is Enough? A Reliable Methodology to Examine Long-Term Wearable Data Acquisition in Gait and Postural Sway. Sensors.

[37] Troiano R.P., McClain J.J., Brychta R.J., Chen K.Y. (2014). Evolution of Accelerometer Methods for Physical Activity Research. Br. J. Sports Med..

[38] Scott J.J., Rowlands A.V., Cliff D.P., Morgan P.J., Plotnikoff R.C., Lubans D.R. (2017). Comparability and Feasibility of Wrist- and Hip-Worn Accelerometers in Free-Living Adolescents. J. Sci. Med. Sport.

[39] McLellan G., Arthur R., Buchan D.S. (2018). Wear Compliance, Sedentary Behaviour and Activity in Free-Living Children from Hip-and Wrist-Mounted ActiGraph GT3X+ Accelerometers. J. Sports Sci..

[40] Goldsack J.C., Coravos A., Bakker J.P., Bent B., Dowling A.V., Fitzer-Attas C., Godfrey A., Godino J.G., Gujar N., Izmailova E. (2020). Verification, Analytical Validation, and Clinical Validation (V3): The Foundation of Determining Fit-for-Purpose for Biometric Monitoring Technologies (BioMeTs). Npj Digit. Med..

[41] Soltani A., Paraschiv-Ionescu A., Dejnabadi H., Marques-Vidal P., Aminian K. (2020). Real-World Gait Bout Detection Using a Wrist Sensor: An Unsupervised Real-Life Validation. IEEE Access.

[42] McDevitt B., Moore L., Akhtar N., Connolly J., Doherty R., Scott W. (2021). Validity of a Novel Research-Grade Physical Activity and Sleep Monitor for Continuous Remote Patient Monitoring. Sensors.

[43] Chan L.L.Y., Choi T.C.M., Lord S.R., Brodie M.A. (2022). Development and Large-Scale Validation of the Watch Walk Wrist-Worn Digital Gait Biomarkers. Sci. Rep..

[44] Femiano R., Werner C., Wilhelm M., Eser P. (2022). Validation of Open-Source Step-Counting Algorithms for Wrist-Worn Tri-Axial Accelerometers in Cardiovascular Patients. Gait Posture.

[45] Mora-Gonzalez J., Gould Z.R., Moore C.C., Aguiar E.J., Ducharme S.W., Schuna J.M., Barreira T.V., Staudenmayer J., McAvoy C.R., Boikova M. (2022). A Catalog of Validity Indices for Step Counting Wearable Technologies during Treadmill Walking: The CADENCE-Adults Study. Int. J. Behav. Nutr. Phys. Act..

[46] Rowlands A.V., Maylor B., Dawkins N.P., Dempsey P.C., Edwardson C.L., Soczawa-Stronczyk A.A., Bocian M., Patterson M.R., Yates T. (2022). Stepping up with GGIR: Validity of Step Cadence Derived from Wrist-Worn Research-Grade Accelerometers Using the Verisense Step Count Algorithm. J. Sports Sci..

[47] Tudor-Locke C., Barreira T.V., Schuna J.M. (2015). Comparison of Step Outputs for Waist and Wrist Accelerometer Attachment Sites. Med. Sci. Sports Exerc..

[48] Nuss K.J., Hulett N.A., Erickson A., Burton E., Carr K., Mooney L., Anderson J., Comstock A., Schlemer E.J., Archambault L.J. (2020). Comparison of Energy Expenditure and Step Count Measured by ActiGraph Accelerometers Among Dominant and Nondominant Wrist and Hip Sites. J. Meas. Phys. Behav..

[49] Activinsights Ltd., GENEActiv Accelerometer. https://activinsights.com/technology/geneactiv/.

[50] Guralnik J.M., Simonsick E.M., Ferrucci L., Glynn R.J., Berkman L.F., Blazer D.G., Scherr P.A., Wallace R.B. (1994). A Short Physical Performance Battery Assessing Lower Extremity Function: Association With Self-Reported Disability and Prediction of Mortality and Nursing Home Admission. J. Gerontol..

[51] Gawel J., Vengrow D., Collins J., Brown S., Buchanan A., Cook C. (2012). The Short Physical Performance Battery as a Predictor for Long Term Disability or Institutionalization in the Community Dwelling Population Aged 65 Years Old or Older. Phys. Ther. Rev..

[52] Pavasini R., Guralnik J., Brown J.C., di Bari M., Cesari M., Landi F., Vaes B., Legrand D., Verghese J., Wang C. (2016). Short Physical Performance Battery and All-Cause Mortality: Systematic Review and Meta-Analysis. BMC Med..

[53] Ramírez-Vélez R., López Sáez De Asteasu M., Morley J.E., Cano-Gutierrez C.A., Izquierdo M. (2021). Performance of the Short Physical Performance Battery in Identifying the Frailty Phenotype and Predicting Geriatric Syndromes in Community-Dwelling Elderly. J. Nutr. Health Aging.

[54] FDA-NIH Biomarker Working Group (2016). BEST (Biomarkers, EndpointS, and Other Tools) Resource.

[55] Esliger D.W., Rowlands A.V., Hurst T.L., Catt M., Murray P., Eston R.G. (2011). Validation of the GENEA Accelerometer. Med. Sci. Sports Exerc..

[56] PAL Technologies Ltd., ActivPAL Accelerometer and PALbatch Desktop Software for Processing of Raw Acceleration Data, Software Version 8.11.1.63. https://www.palt.com/.

[57] Ryan C.G. (2006). The Validity and Reliability of a Novel Activity Monitor as a Measure of Walking. Br. J. Sports Med..

[58] van Hees V.T., Fang Z., Langford J., Assah F., Mohammad A., da Silva I.C.M., Trenell M.I., White T., Wareham N.J., Brage S. (2014). Autocalibration of Accelerometer Data for Free-Living Physical Activity Assessment Using Local Gravity and Temperature: An Evaluation on Four Continents. J. Appl. Physiol..

[59] Fang Z., Langford J., Sweetland C. GENEAread R Package for Reading Binary Files, Version 2.0.9. https://cran.r-project.org/web/packages/GENEAread/index.html.

[60] Campbell C., Gott A., Langford J., Sweetland C. GENEAclassify R Package for the Segmentation and Classification of Accelerometer Data, Version 1.5.2. https://cran.r-project.org/web/packages/GENEAclassify/index.html.

[61] Migueles J.H., Rowlands A.V., Huber F., Sabia S., van Hees V.T. (2019). GGIR: A Research Community–Driven Open Source R Package for Generating Physical Activity and Sleep Outcomes From Multi-Day Raw Accelerometer Data. J. Meas. Phys. Behav..

[62] R Core Team R: A Language and Environment for Statistical Computing, Version 4.1.3. https://www.R-project.org/.

[63] Toth L.P., Park S., Pittman W.L., Sarisaltik D., Hibbing P.R., Morton A.L., Springer C.M., Crouter S.E., Bassett D.R. (2019). Effects of Brief Intermittent Walking Bouts on Step Count Accuracy of Wearable Devices. J. Meas. Phys. Behav..

[64] Stathi A., Withall J., Greaves C.J., Thompson J.L., Taylor G., Medina-Lara A., Green C., Bilzon J., Gray S., Johansen-Berg H. (2018). A Community-Based Physical Activity Intervention to Prevent Mobility-Related Disability for Retired Older People (REtirement in ACTion (REACT)): Study Protocol for a Randomised Controlled Trial. Trials.

[65] Withall J., Greaves C.J., Thompson J.L., de Koning J.L., Bollen J.C., Moorlock S.J., Fox K.R., Western M.J., Snowsill T., Medina-Lara A. (2020). The Tribulations of Trials: Lessons Learnt Recruiting 777 Older Adults Into REtirement in ACTion (REACT), a Trial of a Community, Group-Based Active Aging Intervention Targeting Mobility Disability. J. Gerontol. Ser. A.

[66] Herrmann S.D., Barreira T.V., Kang M., Ainsworth B.E. (2014). Impact of Accelerometer Wear Time on Physical Activity Data: A NHANES Semisimulation Data Approach. Br. J. Sports Med..

[67] Lin L.I.-K. (1989). A Concordance Correlation Coefficient to Evaluate Reproducibility. Biometrics.

[68] Carrasco J.L., King T.S., Chinchilli V.M. (2009). The Concordance Correlation Coefficient for Repeated Measures Estimated by Variance Components. J. Biopharm. Stat..

[69] Carrasco J.L., Martinez J.P. Cccrm: Concordance Correlation Coefficient for Repeated (and Non-Repeated) Measures, Version 2.0.3. https://cran.r-project.org/web/packages/cccrm/index.html.

[70] StataCorp LLC, Stata SE-Standard Edition, Version 17.0 Revision 15 November 2022. http://www.stata.com.

[71] Altman D.G. (1999). Practical Statistics for Medical Research.

[72] Stathi A., Greaves C.J., Thompson J.L., Withall J., Ladlow P., Taylor G., Medina-Lara A., Snowsill T., Gray S., Green C. (2022). Effect of a Physical Activity and Behaviour Maintenance Programme on Functional Mobility Decline in Older Adults: The REACT (Retirement in Action) Randomised Controlled Trial. Lancet Public Health.

[73] Zihajehzadeh S., Park E.J. (2016). Regression Model-Based Walking Speed Estimation Using Wrist-Worn Inertial Sensor. PLoS ONE.

[74] Fasel B., Duc C., Dadashi F., Bardyn F., Savary M., Farine P.-A., Aminian K. (2017). A Wrist Sensor and Algorithm to Determine Instantaneous Walking Cadence and Speed in Daily Life Walking. Med. Biol. Eng. Comput..

[75] Soltani A., Dejnabadi H., Savary M., Aminian K. (2020). Real-World Gait Speed Estimation Using Wrist Sensor: A Personalized Approach. IEEE J. Biomed. Health Inform..

[76] Luu L., Pillai A., Lea H., Buendia R., Khan F.M., Dennis G. (2022). Accurate Step Count with Generalized and Personalized Deep Learning on Accelerometer Data. Sensors.

[77] Ermes M., Parkka J., Mantyjarvi J., Korhonen I. (2008). Detection of Daily Activities and Sports With Wearable Sensors in Controlled and Uncontrolled Conditions. IEEE Trans. Inf. Technol. Biomed..

[78] Gyllensten I.C., Bonomi A.G. (2011). Identifying Types of Physical Activity With a Single Accelerometer: Evaluating Laboratory-Trained Algorithms in Daily Life. IEEE Trans. Biomed. Eng..

[79] Rutjes A., Reitsma J., Coomarasamy A., Khan K., Bossuyt P. (2007). Evaluation of Diagnostic Tests When There Is No Gold Standard. A Review of Methods. Health Technol. Assess..

[80] Kuo P.-L., Urbanek J.K., Schrack J.A. (2019). Age-Related Bias in Total Step Count Recorded by Wearable Devices. JAMA Intern. Med..

[81] Chaudhry U.A.R., Wahlich C., Fortescue R., Cook D.G., Knightly R., Harris T. (2020). The Effects of Step-Count Monitoring Interventions on Physical Activity: Systematic Review and Meta-Analysis of Community-Based Randomised Controlled Trials in Adults. Int. J. Behav. Nutr. Phys. Act..

[82] Guralnik J., Bandeen-Roche K., Bhasin S.A.R., Eremenco S., Landi F., Muscedere J., Perera S., Reginster J.-Y., Woodhouse L., Vellas B. (2019). Clinically meaningful change for physical performance: Perspectives of the icfsr task force. J. Frailty Aging.

[83] Perera S., Mody S.H., Woodman R.C., Studenski S.A. (2006). Meaningful Change and Responsiveness in Common Physical Performance Measures in Older Adults: MEANINGFUL CHANGE AND PERFORMANCE. J. Am. Geriatr. Soc..

[84] Kwon S., Perera S., Pahor M., Katula J.A., King A.C., Groessl E.J., Studenski S.A. (2009). What Is a Meaningful Change in Physical Performance? Findings from a Clinical Trial in Older Adults (the LIFE-P Study). J. Nutr. Health Aging.

[85] Stansfield B., Clarke C., Dall P., Godwin J., Holdsworth R., Granat M. (2015). True Cadence and Step Accumulation Are Not Equivalent: The Effect of Intermittent Claudication on Free-Living Cadence. Gait Posture.

[86] O’Brien M.W., Johns J.A., Frayne R.J., Kimmerly D.S. (2022). Comparison of Habitual Stepping Cadence Analysis Methods: Relationship with Step Counts. Gait Posture.

[87] Hillel I., Gazit E., Nieuwboer A., Avanzino L., Rochester L., Cereatti A., Croce U.D., Rikkert M.O., Bloem B.R., Pelosin E. (2019). Is Every-Day Walking in Older Adults More Analogous to Dual-Task Walking or to Usual Walking? Elucidating the Gaps between Gait Performance in the Lab and during 24/7 Monitoring. Eur. Rev. Aging Phys. Act..

