# Supplementary material for: The Analytical and Clinical Validity of the pfSTEP Digital Biomarker of the Susceptibility/Risk of Declining Physical Function in Community-Dwelling Older Adults
Source: Sensors (Basel). 2023 May 27;23(11):5122. doi: 10.3390/s23115122 (PMC10255880; doi:10.3390/s23115122)
Supplement: Supplementary file 1 [file sensors-23-05122-s001.zip › supplementary_material/Code_S3.rtf]

********************************* Assign variable labels *********************************label define gender 0 "Female" 1 "Male"label value gender genderlabel define site 1 "Bath" 2 "Birmingham" 3 "Exeter"label value site sitelabel define sf36gen_hlth 1 "Very good/Excellent" 2 "Good" 3 "Poor/Fair"label value sf36gen_hlth sf36gen_hlthlabel define comorbid 0 "None" 1 "One or more"label value comorbid comorbidlabel define imd_quintile 1 "Most deprived" 2 "Quite deprived" 3 "Mid deprived" 4 "Little deprived" 5 "Least deprived"label value imd_quintile imd_quintilelabel define highest_education 1 "Some/all secondary" 2 "Some college" 3 "Completed college/degree"label value highest_education highest_educationlabel define allocation 0 "Control" 1 "Intervention"label value allocation allocationlabel define visit 1 "Baseline" 2 "6 Months" 3 "12 Months" 4 "24 Months"label value visit visit***************** Models ****************** mod1 - Total steps only. xtmixed final_sppb_score c.age##i.gender i.site i.sf36gen_hlth i.comorbid i.imd_quintile i.highest_education ///i.allocation##i.visit ///visit_daily_steps_1k c.visit_daily_steps_1k#i.visit ///|| participant_id: , covariance (identity) allbaselevels* Get AIC and BICestat ic* Predict standardised residualspredict preds_mod1predict resids_mod1, rstandard* Scatter plot of predicted SPPB scores and residualsscatter resids_mod1 preds_mod1* QQ-Plotqnorm resids_mod1* mod2 - Faster walking steps only. xtmixed final_sppb_score c.age##i.gender i.site i.sf36gen_hlth i.comorbid i.imd_quintile i.highest_education ///i.allocation##i.visit ///visit_faster_steps_1k c.visit_faster_steps_1k#i.visit ///|| participant_id: , covariance (identity) allbaselevels* Get AIC and BICestat ic* Predict standardised residualspredict preds_mod2predict resids_mod2, rstandard* Scatter plot of predicted SPPB scores and residualsscatter resids_mod2 preds_mod2* QQ-Plotqnorm resids_mod2* mod3 - Non-fast steps plus faster walking steps. xtmixed final_sppb_score c.age##i.gender i.site i.sf36gen_hlth i.comorbid i.imd_quintile i.highest_education ///i.allocation##i.visit ///visit_non_fast_steps_1k visit_faster_steps_1k c.visit_non_fast_steps_1k#i.visit c.visit_faster_steps_1k#i.visit ///|| participant_id: , covariance (identity) allbaselevels* Get AIC and BICestat ic* Predict standardised residualspredict preds_mod3predict resids_mod3, rstandard* Scatter plot of predicted SPPB scores and residualsscatter resids_mod3 preds_mod3* QQ-Plotqnorm resids_mod3******************************* Sensitivity analysis ********************************** Without health and social status covariates **** mod1b - Total steps only. mixed final_sppb_score c.age##i.gender i.site ///i.allocation##i.visit ///visit_daily_steps_1k c.visit_daily_steps_1k#i.visit ///|| participant_id: , covariance (identity) allbaselevels* Get AIC and BICestat ic* Predict standardised residualspredict preds_mod1bpredict resids_mod1b, rstandard* Scatter plot of predicted SPPB scores and residualsscatter resids_mod1b preds_mod1b* QQ-Plotqnorm resids_mod1b* mod2b - Faster walking steps only. mixed final_sppb_score c.age##i.gender i.site ///i.allocation##i.visit ///visit_faster_steps_1k c.visit_faster_steps_1k#i.visit ///|| participant_id: , covariance (identity) allbaselevels* Get AIC and BICestat ic* Predict standardised residualspredict preds_mod2bpredict resids_mod2b, rstandard* Scatter plot of predicted SPPB scores and residualsscatter resids_mod2b preds_mod2b* QQ-Plotqnorm resids_mod2b* mod3b - Non-fast steps plus faster walking steps. mixed final_sppb_score c.age##i.gender i.site ///i.allocation##i.visit ///visit_non_fast_steps_1k visit_faster_steps_1k c.visit_non_fast_steps_1k#i.visit c.visit_faster_steps_1k#i.visit ///|| participant_id: , covariance (identity) allbaselevels* Get AIC and BICestat ic* Predict standardised residualspredict preds_mod3bpredict resids_mod3b, rstandard* Scatter plot of predicted SPPB scores and residualsscatter resids_mod3b preds_mod3b* QQ-Plotqnorm resids_mod3b*** Ordinal logistic regression with all covariates **** mod1c - Total steps only. meologit final_sppb_score c.age##i.gender i.site i.sf36gen_hlth i.comorbid i.imd_quintile i.highest_education ///i.allocation##i.visit ///visit_daily_steps_1k c.visit_daily_steps_1k#i.visit ///|| participant_id: , covariance (identity) allbaselevels* Get AIC and BICestat ic* mod2c - Faster walking steps only. meologit final_sppb_score c.age##i.gender i.site i.sf36gen_hlth i.comorbid i.imd_quintile i.highest_education ///i.allocation##i.visit ///visit_faster_steps_1k c.visit_faster_steps_1k#i.visit ///|| participant_id: , covariance (identity) allbaselevels* Get AIC and BICestat ic* mod3c - Non-fast steps plus faster walking steps. meologit final_sppb_score c.age##i.gender i.site i.sf36gen_hlth i.comorbid i.imd_quintile i.highest_education ///i.allocation##i.visit ///visit_non_fast_steps_1k visit_faster_steps_1k c.visit_non_fast_steps_1k#i.visit c.visit_faster_steps_1k#i.visit ///|| participant_id: , covariance (identity) allbaselevels* Get AIC and BICestat ic***************************** Control group only ****************************** mod1d - Total steps only. mixed final_sppb_score c.age##i.gender i.site i.sf36gen_hlth i.comorbid i.imd_quintile i.highest_education i.visit ///visit_daily_steps_1k c.visit_daily_steps_1k#i.visit ///if allocation == 0 ///|| participant_id: , covariance (identity) allbaselevels* Get AIC and BICestat ic* Predict standardised residualspredict preds_mod1dpredict resids_mod1d, rstandard* Scatter plot of predicted SPPB scores and residualsscatter resids_mod1d preds_mod1d* QQ-Plotqnorm resids_mod1d* mod2d - Faster walking steps only. mixed final_sppb_score c.age##i.gender i.site i.sf36gen_hlth i.comorbid i.imd_quintile i.highest_education i.visit ///visit_faster_steps_1k c.visit_faster_steps_1k#i.visit ///if allocation == 0 ///|| participant_id: , covariance (identity) allbaselevels* Get AIC and BICestat ic* Predict standardised residualspredict preds_mod2dpredict resids_mod2d, rstandard* Scatter plot of predicted SPPB scores and residualsscatter resids_mod2d preds_mod2d* QQ-Plotqnorm resids_mod2d* mod3d - Non-fast steps plus faster walking steps. mixed final_sppb_score c.age##i.gender i.site i.sf36gen_hlth i.comorbid i.imd_quintile i.highest_education i.visit ///visit_non_fast_steps_1k visit_faster_steps_1k c.visit_non_fast_steps_1k#i.visit c.visit_faster_steps_1k#i.visit ///if allocation == 0 ///|| participant_id: , covariance (identity) allbaselevels* Get AIC and BICestat ic* Predict standardised residualspredict preds_mod3dpredict resids_mod3d, rstandard* Scatter plot of predicted SPPB scores and residualsscatter resids_mod3d preds_mod3d* QQ-Plotqnorm resids_mod3d********************************** Intervention group only *********************************** mod1e - Total steps only. mixed final_sppb_score c.age##i.gender i.site i.sf36gen_hlth i.comorbid i.imd_quintile i.highest_education i.visit ///visit_daily_steps_1k c.visit_daily_steps_1k#i.visit ///if allocation == 1 ///|| participant_id: , covariance (identity) allbaselevels* Get AIC and BICestat ic* Predict standardised residualspredict preds_mod1epredict resids_mod1e, rstandard* Scatter plot of predicted SPPB scores and residualsscatter resids_mod1e preds_mod1e* QQ-Plotqnorm resids_mod1e* mod2e - Faster walking steps only. mixed final_sppb_score c.age##i.gender i.site i.sf36gen_hlth i.comorbid i.imd_quintile i.highest_education i.visit ///visit_faster_steps_1k c.visit_faster_steps_1k#i.visit ///if allocation == 1 ///|| participant_id: , covariance (identity) allbaselevels* Get AIC and BICestat ic* Predict standardised residualspredict preds_mod2epredict resids_mod2e, rstandard* Scatter plot of predicted SPPB scores and residualsscatter resids_mod2e preds_mod2e* QQ-Plotqnorm resids_mod2e* mod3e - Non-fast steps plus faster walking steps. mixed final_sppb_score c.age##i.gender i.site i.sf36gen_hlth i.comorbid i.imd_quintile i.highest_education i.visit ///visit_non_fast_steps_1k visit_faster_steps_1k c.visit_non_fast_steps_1k#i.visit c.visit_faster_steps_1k#i.visit ///if allocation == 1 ///|| participant_id: , covariance (identity) allbaselevels* Get AIC and BICestat ic* Predict standardised residualspredict preds_mod3epredict resids_mod3e, rstandard* Scatter plot of predicted SPPB scores and residualsscatter resids_mod3e preds_mod3e* QQ-Plotqnorm resids_mod3e
